# Runx2 Regulates *Galnt3* and *Fgf23* Expressions and Galnt3 Decelerates Osteoid Mineralization by Stabilizing Fgf23

**DOI:** 10.3390/ijms25042275

**Published:** 2024-02-14

**Authors:** Qing Jiang, Xin Qin, Takeshi Moriishi, Ryo Fukuyama, Shinichi Katsumata, Hiroshi Matsuzaki, Hisato Komori, Yuki Matsuo, Chiharu Sakane, Kosei Ito, Hironori Hojo, Shinsuke Ohba, Toshihisa Komori

**Affiliations:** 1 Institute of Orthopaedics, Suzhou Medical College, Soochow University, Suzhou 215006, China; 2 Department of Molecular Bone Biology, Nagasaki University Graduate School of Biomedical Sciences, Nagasaki 852-8588, Japan; 3 Department of Cell Biology, Nagasaki University Graduate School of Biomedical Sciences, Nagasaki 852-8588, Japan; 4 Laboratory of Pharmacology, Hiroshima International University, Kure 737-0112, Japan; 5 Department of Nutritional Science, Faculty of Applied Bioscience, Tokyo University of Agriculture, Tokyo 156-8502, Japan; 6 Research Center for Biomedical Models and Animal Welfare, Nagasaki University, Nagasaki 852-8523, Japan; 7 Laboratory of Clinical Biotechnology, Center for Disease Biology and Integrative Medicine, Graduate School of Medicine, The University of Tokyo, Tokyo 113-8655, Japan; 8 Department of Tissue and Developmental Biology, Graduate School of Dentistry, Osaka University, Osaka 565-0871, Japan

**Keywords:** Runx2, Galnt3, Fgf23, osteocyte, phosphorus, hyperphosphatemia, osteoid, mineralization

## Abstract

Runx2 (runt related transcription factor 2) is an essential transcription factor for osteoblast proliferation and differentiation. Uridine diphosphate (UDP)-N-acetylgalactosamine (GalNAc): polypeptide GalNAc-transferase 3 (Galnt3) prevents proteolytic processing of fibroblast growth factor 23 (Fgf23), which is a hormone that regulates the serum level of phosphorus. *Runx2* and *Galnt3* were expressed in osteoblasts and osteocytes, and *Fgf23* expression was restricted to osteocytes in bone. Overexpression and knock-down of *Runx2* upregulated and downregulated, respectively, the expressions of *Galnt3* and *Fgf23*, and Runx2 directly regulated the transcriptional activity of *Galnt3* in reporter assays. The expressions of *Galnt3* and *Fgf23* in osteoblast-specific *Runx2* knockout (*Runx2*^fl/flCre^) mice were about half those in *Runx2*^fl/fl^ mice. However, the serum levels of phosphorus and intact Fgf23 in *Runx2*^fl/flCre^ mice were similar to those in *Runx2*^fl/fl^ mice. The trabecular bone volume was increased during aging in both male and female *Galnt3*^−/−^ mice, but the osteoid was reduced. The markers for bone formation and resorption in *Galnt3*^−/−^ mice were similar to the control in both sexes. *Galnt3*^−/−^ mice exhibited hyperphosphatemia and hypercalcemia, and the intact Fgf23 was about 40% that of wild-type mice. These findings indicated that Runx2 regulates the expressions of *Galnt3* and *Fgf23* and that Galnt3 decelerates the mineralization of osteoid by stabilizing Fgf23.

## 1. Introduction

Mesenchymal stem cells differentiate into chondrocytes, osteoblasts, adipocytes, muscle cells, and tendon cells, and each differentiation is regulated by specific transcription factors. Runx2 is an essential transcription factor for osteoblast differentiation [1]. Runx2 induces the commitment of multipotent mesenchymal cells into osteoblast lineage cells, enhances the proliferation of osteoblast progenitors, and induces the expressions of major bone matrix protein genes in osteoblasts [1]. Runx2 is also required for chondrocyte proliferation and maturation [1,2]. We introduced *Runx2* into *Runx2*^−/−^ chondrocytes and searched Runx2 target genes by microarray analysis [3]. *Galnt3* was upregulated by Runx2, and Galnt3 was involved in chondrocyte maturation [3]. Although Galnt3 is also expressed in osteoblasts, the regulation of *Galnt3* expression by Runx2 in osteoblasts and the physiological role of the Runx2–Galnt3 axis in bone development and maintenance remains to be clarified. 

*Galnt3* encodes UDP-N-acetylgalactosamine (GalNAc): polypeptide GalNAc-transferase 3 (Galnt3), which catalyzes the first step of mucin-type O-glycosylation forming the GalNAcα1-O-serine (Ser)/threonine (Thr) linkage in O-glycoproteins [4]. Mutations in *GALNT3* cause the related diseases, familial tumoral calcinosis, which is characterized by hyperphosphatemia and ectopic calcifications around major joints, and hyperostosis-hyperphosphatemia syndrome, which is characterized by hyperphosphatemia and recurrent long bone lesions with hyperostosis [5,6,7,8]. The *GALNT3* mutations cause impaired O-linked glycosylation of FGF23, which leads to the proteolytic processing of FGF23. Fgf23, which is expressed in osteocytes [9], inhibits reabsorption of phosphate in kidney and renal biosynthesis of 1, 25(OH)_2_D_3_, and reduces the serum level of phosphorus [10].

*Galnt3*-deficient (*Galnt3*^−/−^) male mice show retarded growth, infertility, and increases in trabecular bone volume and cortical bone area, while *Galnt3*^−/−^ female mice show normal growth and no bone phenotypes [11], although both male and female *Galnt3*^−/−^ mice show hyperphosphatemia and reduction in the serum levels of intact Fgf23 [12]. *Galnt3*^−/−^ male infertility is caused by impaired acrosome formation [13]. We previously reported that Runx2 induces *Galnt3* expression in chondrocytes [3]. As Galnt3 regulates the stability of Fgf23, we examined the regulation of *Galnt3* expression by Runx2, the involvement of Runx2 in phosphorus homeostasis, and the role of Galnt3 in bone development and maintenance.

## 2. Results

### 2.1. Runx2 and Galnt3 Are Expressed in Osteoblasts and Osteocytes, and Fgf23 Is Expressed in Osteocytes

**Figure 1:**
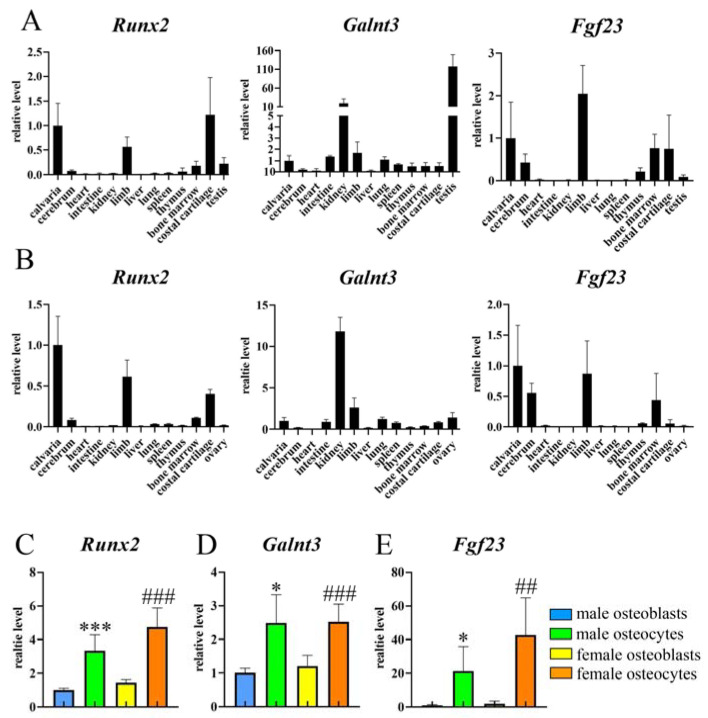
The expressions of *Runx2*, *Glant3*, and *Fgf23* in mice. (**A**,**B**) Real-time RT-PCR analysis of *Runx2*, *Glant3*, and *Fgf23* expressions using RNA from tissues, including calvaria, cerebrum, heart, intestine, limb, liver, lung, spleen, thymus, bone marrow, costal cartilage, testis, and ovary in male (**A**) and female (**B**) wild-type mice at 4 weeks of age. The values in calvaria were defined as 1, and relative levels are shown. The samples were prepared from four mice for each tissue. (**C**–**E**) The expressions of *Runx2*, *Galnt3*, and *Fgf23* in osteoblast-enriched fractions and osteocyte-enriched fractions from femurs and tibiae in male and female wild-type mice at 9 weeks of age. The values in male osteoblast-enriched fractions were defined as 1, and relative levels are shown. n = 6. * vs. male osteoblast-enriched fractions, # vs. female osteoblast-enriched fractions. * *p* < 0.05, ## *p* < 0.01, ***, ### *p* < 0.001.

First, we examined mRNA expression of *Runx2*, *Galnt3*, and *Fgf23* in many tissues of male and female wild-type mice. Indeed, Runx2 expression was high in skeletal tissues, including calvaria, limb, and costal cartilage, of both sexes (Figure 1A,B). *Galnt3* expression was extremely high in testis (Figure 1A). The second highly expressed tissue was kidney, and the expression in calvaria and limb was relatively high among the other tissues in both sexes (Figure 1A,B). *Fgf23* was expressed highly in calvaria and limb and mildly in cerebrum and bone marrow of both sexes (Figure 1A,B). In real-time RT-PCR using the osteoblast-enriched and osteocyte-enriched fractions, the relative expression of *Runx2* and *Galnt3* against *Actb* in the osteocyte-enriched fraction was higher than that in the osteoblast-enriched fraction in both sexes (Figure 1C,D), while that of *Fgf23* was extremely higher in the osteocyte-enriched fraction than the osteoblast-enriched fraction in both sexes (Figure 1E). Thus, these findings indicated that *Runx2* and *Galnt3* are expressed in osteoblasts and osteocytes and *Fgf23* is expressed in osteocytes in bone tissues. 

### 2.2. Runx2 Regulates Galnt3 and Fgf23 Expressions in Osteoblast Lineage Cells

**Figure 2:**
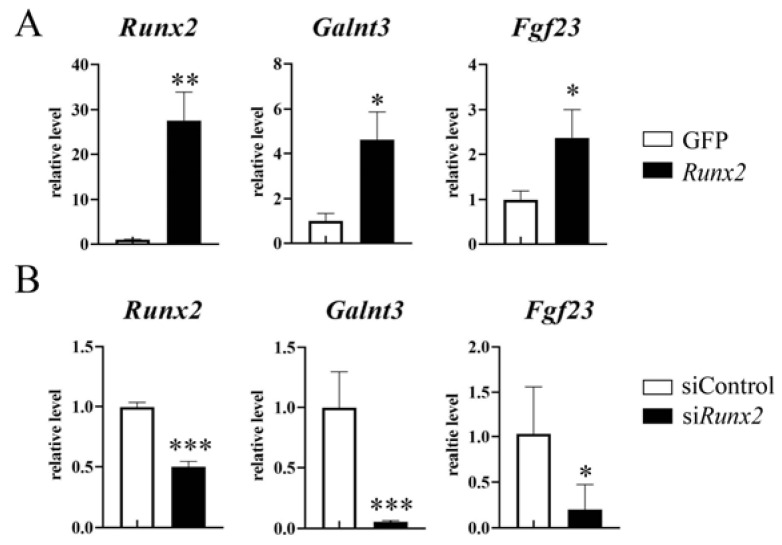
Regulation of *Galnt3* and *Fgf23* expressions by Runx2. (**A**) Induction of *Glant3* and *Fgf23* expressions by Runx2. Primary osteoblasts from wild-type newborns were infected with an adenovirus expressing green fluorescent protein (GFP) or *Runx2*-GFP, and *Runx2*, *Glant3*, and *Fgf23* expressions were analyzed by real-time RT-PCR. The values in GFP-expressing cells were defined as 1, and relative levels are shown. n = 4. * vs. GFP. * *p* < 0.05, ** *p* < 0.01. (**B**) Primary osteoblasts were transfected with siRNA for control (siControl) or Runx2 (si*Runx2*), and RNA was extracted after the culture for 7 days in osteogenic medium. The expressions of *Runx2*, *Galnt3*, and *Fgf23* were examined by real-time RT-PCR. The values in siControl were defined as 1, and relative levels are shown. n = 5. * vs. siControl. * *p* < 0.05, *** *p* < 0.001.

To examine whether Runx2 regulates *Galnt3* and *Fgf23* expressions in osteoblasts, *Runx2* was overexpressed in primary osteoblasts from wild-type newborns using adenovirus (Figure 2A). Overexpression of *Runx2* induced *Galnt3* and *Fgf23* expressions. Further, introduction of *Runx2* siRNA into wild-type primary osteoblasts reduced the expressions of *Runx2*, *Galnt3*, and *Fgf23* (Figure 2B). These findings suggest that Runx2 regulates the expressions of *Galnt3* and *Fgf23* in osteoblast-lineage cells. 

### 2.3. Runx2 Directly Regulates Galnt3 Expression

**Figure 3:**
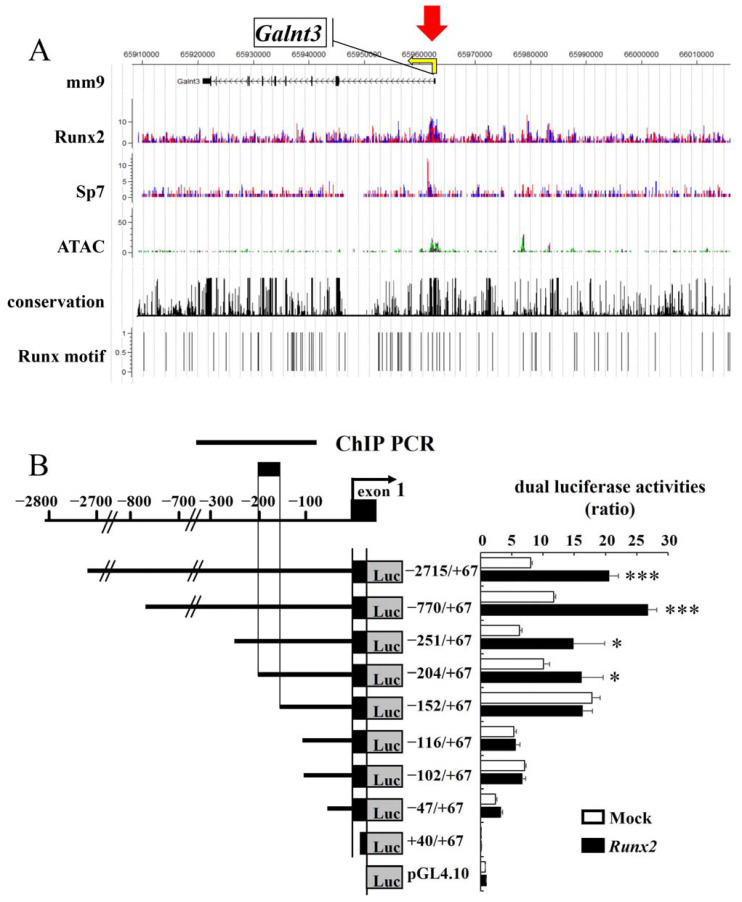
ChIP-seq and ATAC-seq in *Galnt3* locus and reporter assays in *Galnt3* promoter region. (**A**) CisGenome browser screenshot of the flanking region of *Galnt3* showing the association of Runx2 and Sp7 and chromatin accessibility (ATAC) in Sp7-positive primary osteoblasts. Sp7 was included as reference data. The sequence conservation and Runx motif mapping are shown together. The scale indicates the intensity of enrichment. Red arrow indicates the peak. (**B**) Reporter assays using a reporter construct containing 2.8 kb DNA fragment of *Galnt3* promoter region and the deletion constructs. The region responsible for the activation by Runx2 is shown by a thick bar. ChIP PCR shows the region, which was amplified in ChIP assay in Figure 4C. * vs. Mock. * *p* < 0.05, *** *p* < 0.001.

**Figure 4:**
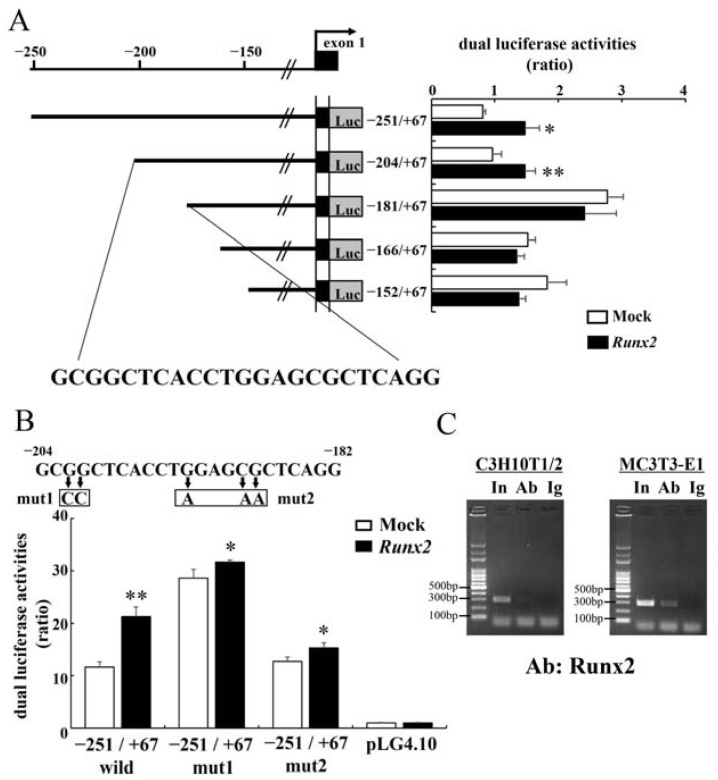
Reporter and ChIP assays in *Galnt3* promoter region. (**A**) Reporter assays using a reporter construct containing −251/+67 fragment and the deletion constructs in *Galnt3* promoter region. The 23 bp is a candidate sequence for Runx2 binding. (**B**) Site-specific mutagenesis of putative Runx2 binding sites in the 23 bp. The mutated nucleotides are shown in the boxes. In all reporter assays, C3H10T1/2 cells were transfected with an empty (open column) or *Runx2*-expressing (closed column) vector. * vs. Mock. * *p* < 0.05, ** *p* < 0.01. (**C**) ChIP assays. DNA before (input: In) and after immunoprecipitation with a monoclonal anti-Runx2 antibody (Ab) or mouse IgG (Ig) was amplified by PCR using primers that amplify the DNA fragment shown as ChIP PCR in Figure 3B.

We analyzed chromatin immunoprecipitation sequencing (ChIP-seq) data using primary osteoblasts in the flanking regions of *Galnt3* and *Fgf23*. ChIP-seq showed the association of Runx2 in the promoter region of *Galnt3*, which has a peak in assay for transposase-accessible chromatin with high-throughput sequencing (ATAC-seq) using Sp7-positive primary osteoblasts (Figure 3A). There were a few peaks of Runx2 and ATAC-seq distant from *Fgf23*, and the peak of ATAC-seq in the promoter region of *Fgf23* was low (Supplementary Figure S1). *Fgf23* was expressed in osteocytes (Figure 1E), but the database of ChIP-seq and ATAC-seq, which we analyzed, had been obtained using primary osteoblasts. Due to the lack of a database using osteocytes, it was difficult to identify Runx2 binding regions responsible for the induction of *Fgf23* expression. Thus, we focused on the promoter region of *Galnt3* and performed reporter assay of 2.8 kb DNA fragment covering the promoter region of *Galnt3* using C3H10T1/2 cells. Introduction of *Runx2* enhanced the reporter activity of −2715/+67 construct, and it enhanced the activities of the deletion constructs, including −770/+67, −251/+67, and −204/+67 (Figure 3B). However, *Runx2* failed to enhance the activities of –152/+67 construct and further deleted constructs, including −116/+67, −102/+67, −47/+67, and +40/+67 (Figure 3B). Therefore, we focused on the sequences between −204 and −152. The deletion of −204/+67 construct to −181/+67, –166/+67, −152/+67 constructs abolished the activation by *Runx2* (Figure 4A). There was no ideal Runx2 binding sequence (PyGPyGGTPy) in the 23 bp from −204 to −182, and four base matched sequences with the consensus sequence were GCGGCT, ACCTGG, and AGCGCT (Figure 4A). We generated −251/+67 constructs with mutation 1 or mutation 2 as shown in Figure 4B. Either mutation 1 or 2 mostly abolished the transcriptional activation by Runx2 (Figure 4B). The binding of Runx2 in this region was confirmed by ChIP using C3H10T1/2 and MC3T3-E1 cells (Figure 4C).

### 2.4. The Expressions of Galnt3 and Fgf23 Were Reduced in Runx2^fl/flCre^ Mice

**Figure 5:**
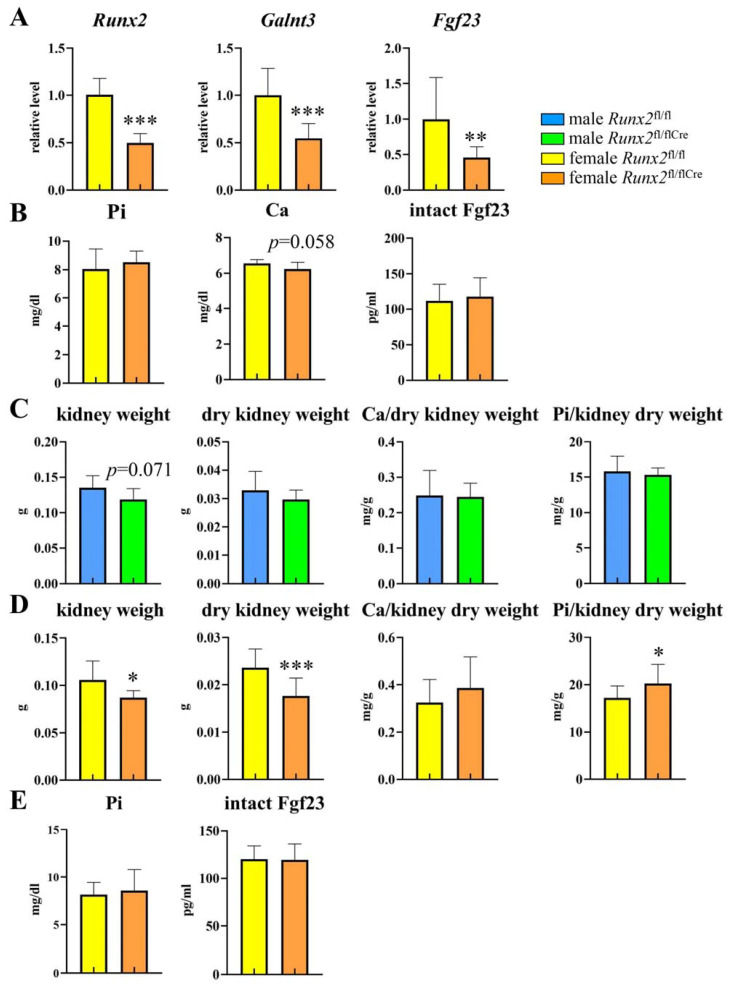
Homeostasis of phosphorus in *Runx2*^fl/flCre^ mice. (**A**) Real-time RT-PCR analysis of *Runx2*, *Galnt3*, and *Fgf23* expression in female *Runx2*^fl/flCre^ mice. RNA was extracted from tibiae at 6 weeks of age. The values in *Runx2*^fl/fl^ mice were defined as 1, and relative levels are shown. n = 16 (*Runx2*^fl/fl^), n = 8 (*Runx2*^fl/flCre^). (**B**) Serum levels of phosphorus (Pi), calcium (Ca), and intact Fgf23 in female *Runx2*^fl/flCre^ mice at 6 weeks of age. n = 15 (*Runx2*^fl/fl^), n = 8 (*Runx2*^fl/flCre^). (**C**,**D**) Weights of wet and dry kidneys, and the ratios of Ca and Pi to the dry kidney weight in male (**C**) and female (**D**) *Runx2*^fl/fl^ and *Runx2*^fl/flCre^ mice with high phosphate diet. n = 8 (*Runx2*^fl/fl^), n = 7 (*Runx2*^fl/flCre^) in C, n = 11 (*Runx2*^fl/fl^), n = 17 (*Runx2*^fl/flCre^) in D. (**E**) Serum Pi and intact Fgf23 in the mice analyzed in D. * vs. *Runx2*^fl/fl^ mice. * *p* < 0.05, ** *p* < 0.01, *** *p* < 0.001.

We examined the expressions of *Runx2*, *Galnt3*, and *Fgf23* and the serum levels of phosphorus, calcium, and intact Fgf23 in female *Runx2* conditional knockout (*Runx2*^fl/flCre^) mice, which were generated using 2.3 kb *Col1a1* GFP-Cre transgenic mice, at 6 weeks of age. The expressions of *Runx2*, *Galnt3*, and *Fgf23* in *Runx2*^fl/flCre^ mice were about half those in *Runx2*^fl/fl^ mice (Figure 5A). The serum levels of phosphorus, calcium, and intact Fgf23 in *Runx2*^fl/flCre^ mice were comparable to those in *Runx2*^fl/fl^ mice (Figure 5B). As the serum phosphorus level was not increased in *Runx2*^fl/flCre^ mice irrespective of the reductions in *Galnt3* and *Fgf23* expressions, the phosphorus content in dry kidney after a high phosphorus diet was examined to evaluate the phosphorus homeostasis. *Runx2*^fl/fl^ mice and *Runx2*^fl/flCre^ mice at 3 weeks of age were fed on a high phosphorus diet for 4 weeks. At 7 weeks of age, they were sacrificed, and the wet kidney weight, dry kidney weight, and the contents of calcium and phosphorus in the dry kidneys were measured. The wet and dry kidney weights in *Runx2*^fl/flCre^ mice were lower in females but not in males than those in *Runx2*^fl/fl^ mice (Figure 5C,D). Although the ratios of the calcium to the dry kidney weight were similar between *Runx2*^fl/fl^ and *Runx2*^fl/flCre^ mice in males and females, the ratio of the phosphorus to the dry kidney weight in *Runx2*^fl/flCre^ mice was higher than that in *Runx2*^fl/fl^ mice in females but not in males (Figure 5C,D). However, the serum levels of phosphorus and intact Fgf23 were similar between female *Runx2*^fl/fl^ and *Runx2*^fl/flCre^ mice (Figure 5E).

### 2.5. Trabecular Bone Volume in Galnt3^−/−^ Mice Was Higher Than That in Wild-Type Mice in Both Males and Females at 30 Weeks of Age

**Figure 6:**
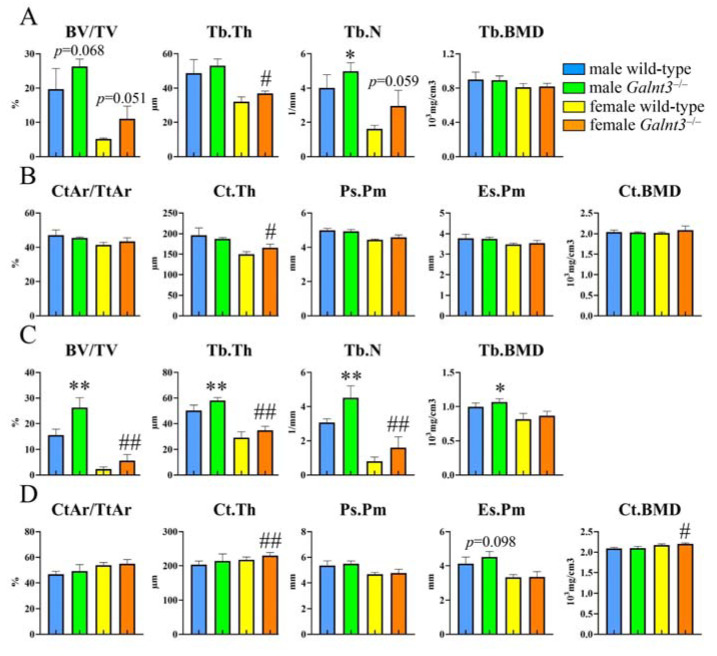
Micro-CT analyses of femurs in *Galnt3*^−/−^ mice. Quantification of the trabecular bone volume (BV/TV), trabecular thickness (Tb.Th), trabecular number (Tb.N), and trabecular bone mineral density (Tb. BMD) in the trabecular bone of distal femoral metaphysis at 8 weeks (**A**) and 30 weeks of age (**C**). Quantification of the cortical area (CtAr/TtAr), cortical thickness (Ct.Th), periosteal perimeter (Ps.Pm), endosteal perimeter (Es.Pm), and cortical bone mineral density (Ct. BMD) in the mid-diaphysis of cortical bone in femurs at 8 weeks (**B**) and 30 weeks of age (**D**). n = 3–5 (**A**,**B**), n = 5–6 (male mice in C and D), n = 9–10 (female mice in C and D). * vs. wild-type males, # vs. wild-type females. *, # *p* < 0.05, **, ## *p* < 0.01.

As *Galnt3* was a direct target of Runx2, bone phenotypes of *Galnt3*^−/−^ mice were analyzed. The body weights of male but not female *Galnt3*^−/−^ mice were reduced compared with those of wild-type mice (Supplementary Figure S2), and no ectopic calcification was observed, as previously reported [11]. Contrary to the previous report that showed reduced body weights in male *Galnt3*^+/−^ mice, however, the body weights of male *Galnt3*^+/−^ mice were like those in wild-type mice at least until 6 weeks of age. The previous report showed that the bone mineral densities (BMDs) in whole body, legs, and extracted femurs in *Galnt3*^−/−^ mice are higher than those in wild-type mice in males but not in females, and the female mice were not analyzed in detail [11]. To investigate whether male and female *Galnt3*^−/−^ mice show different bone phenotypes, we performed micro-CT analysis in both males and females at 8 and 30 weeks of age (Figure 6). At 8 weeks of age, the trabecular bone volume and BMD in *Galnt3*^−/−^ mice were not significantly different from those in wild-type mice in both males and females, although the trabecular thickness in female *Galnt3*^−/−^ mice and trabecular number in male *Galnt3*^−/−^ mice were higher than that in the respective wild-type mice (Figure 6A). The cortical thickness in *Galnt3*^−/−^ mice was greater than that in wild-type mice in females but not in males, and the other parameters, including cortical area, periosteal perimeter, endosteal perimeter, and cortical BMD, were similar in wild-type and *Galnt3*^−/−^ mice in both males and females (Figure 6B). 

**Figure 7:**
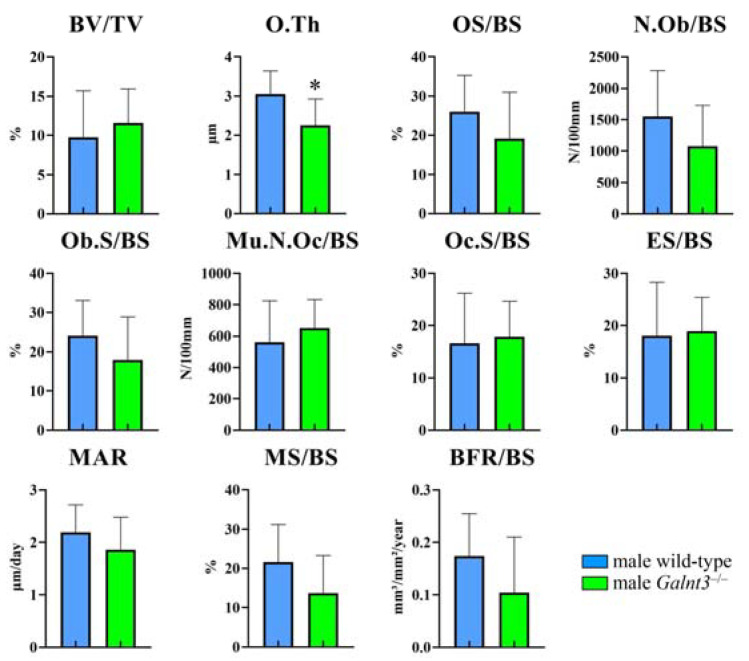
Bone histomorphometric analysis in *Galnt3*^−/−^ mice. The trabecular bone volume (BV/TV), osteoid thickness (O.Th), osteoid surface (OS/BS), number of osteoblasts (N.Ob/BS), osteoblast surface (Ob.S/BS), number of multinucleated osteoclasts (Mu.N.Oc/BS), osteoclast surface (Oc.S/BS), eroded surface (ES/BS), mineral apposition rate (MAR), mineralizing surface (MS/BS), and bone formation rate (BFR/BS) were analyzed in the trabecular bone in femurs of male wild-type and *Galnt3*^−/−^ mice at 8 weeks of age. BS: bone surface. n = 7–10. * vs. wild-type mice. * *p* < 0.05.

At 30 weeks of age, the trabecular bone volume, trabecular thickness, and trabecular number in *Galnt3*^−/−^ mice were higher than those in wild-type mice in both males and females, but trabecular BMD in *Galnt3*^−/−^ mice was higher than that in wild-type mice in males but not in females (Figure 6C). All parameters in cortical bone were similar between male wild-type and *Galnt3*^−/−^ mice, whereas the cortical thickness and cortical BMD in female *Galnt3*^−/−^ mice were greater than those in female wild-type mice. The other cortical parameters were similar between female wild-type and *Galnt3*^−/−^ mice (Figure 6D). Thus, an increase of the trabecular bone volume in *Galnt3*^−/−^ mice was apparent at 30 weeks of age in both males and females, and the cortical thickness was increased in only female *Galnt3*^−/−^ mice at 8 and 30 weeks of age. In bone histomorphometric analysis of trabecular bone in male mice at 8 weeks of age, the osteoid thickness in *Galnt3*^−/−^ mice was less than that in wild-type mice (Figure 7). The other parameters, including osteoid surface, osteoblast number, osteoblast surface, osteoclast number, osteoclast surface, eroded surface, mineral apposition rate, mineralizing surface, and bone formation rate, in *Galnt3*^−/−^ mice were comparable to those in wild-type mice (Figure 7).

### 2.6. The Serum Levels of Osteocalcin and TRAP5b Were Similar between Wild-Type and Galnt3^−/−^ Mice, Those of Phosphorus and Calcium Were Increased, and Those of Intact Fgf23 Were Reduced in Both Males and Females

**Figure 8:**
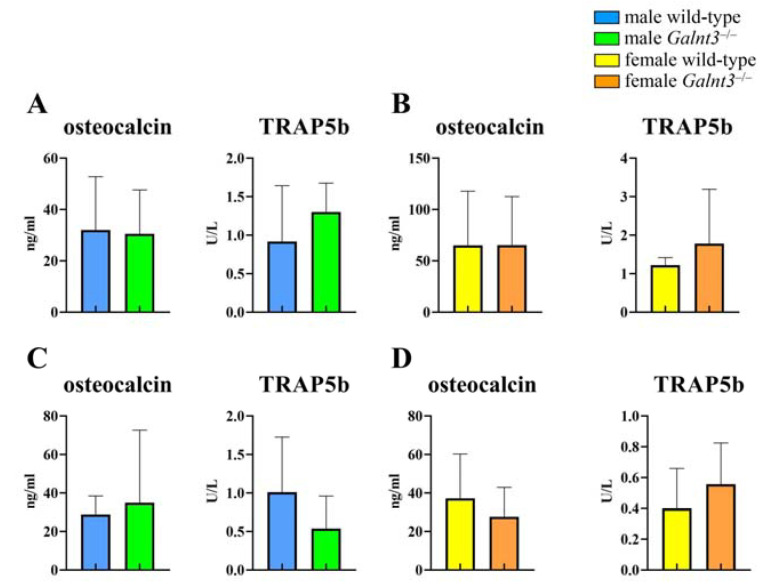
Serum levels of osteocalcin and TRAP5b in *Galnt3*^−/−^ mice. Serum levels of osteocalcin and TRAP5b in male (**A**,**C**) and female (**B**,**D**) wild-type and *Galnt3*^−/−^ mice at 8 weeks of age (**A**,**B**) and 30 weeks of age (**C**,**D**). n = 5 in A, n = 3–4 in B, n = 3–6 in C, and n = 8–10 in D.

**Figure 9:**
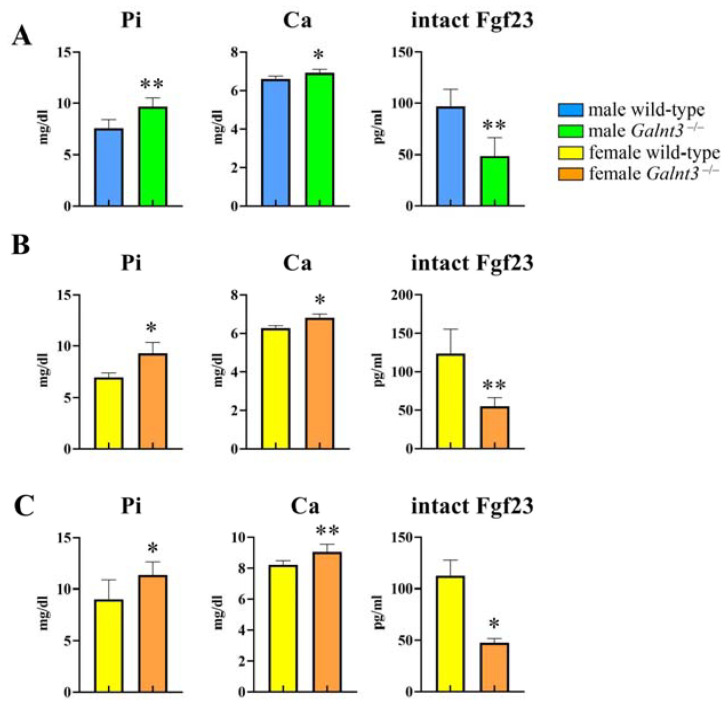
Serum levels of Pi, Ca, and intact Fgf23 in *Galnt3*^−/−^ mice. (**A**–**C**) Serum levels of Pi, Ca, and intact Fgf23 in wild-type and *Galnt3*^−/−^ mice in males (**A**) and females (**B**) at 8 weeks of age and females at 17 weeks of age (**C**). n = 3–5 (**A**), n = 3–4 (**B**), n = 3–7 (**C**). * vs. wild-type mice. * *p* < 0.05, ** *p* < 0.01.

The serum levels of the bone formation marker, osteocalcin, and bone resorption marker, tartrate resistant acid phosphatase 5b (TRAP5b), were examined at 8 and 30 weeks of age. The serum levels of osteocalcin and TRAP5b were similar between wild-type and *Galnt3*^−/−^ mice at 8 and 30 weeks of age in both males and females (Figure 8). The serum levels of phosphorus and calcium were increased and those of intact Fgf23 were reduced in *Galnt3*^−/−^ mice at 8 weeks of age similarly in males and females (Figure 9A,B) and at 17 weeks of age in females (Figure 9C) compared with those in the respective wild-type mice.

## 3. Discussion

*Runx2* and *Galnt3* were expressed in osteoblasts and osteocytes, and *Fgf23* was expressed in osteocytes in bone. Runx2 regulated the expressions of *Galnt3* and *Fgf23*, the levels of *Galnt3* and *Fgf23* mRNA in *Runx2*^fl/flCre^ mice were about half those in *Runx2*^fl/fl^ mice, and Runx2 directly regulated *Galnt3* transcription. However, the levels of serum phosphorus and intact Fgf23 in *Runx2*^fl/flCre^ mice were comparable to those in *Runx2*^fl/fl^ mice. Thus, Runx2 regulated *Galnt3* and *Fgf23* expressions, but Runx2 was not essential for the homeostasis of serum phosphorus in a physiological condition. The absence of *Galnt3* increased trabecular bone volume during aging in both males and females without affecting bone formation and resorption. 

Although the expressions of both *Galnt3* and *Fgf23* in *Runx2*^fl/flCre^ mice were about half those in *Runx2*^fl/fl^ mice, the serum level of intact *Fgf23* in *Runx2*^fl/flCre^ mice was not reduced. Although *Fgf23* mRNA expression in the femurs in *Galnt3*^−/−^ mice was about three times higher than that in wild-type mice, the serum level of total Fgf23 protein including the C-terminal fragments in *Galnt3*^−/−^ mice was about 16 times higher than that in wild-type mice [14]. Thus, the total *Fgf23* protein is likely to be regulated by the serum level of phosphorus via a feedback loop not only at the transcription level but also at the translation level. It may explain why the serum intact *Fgf23* and phosphorus were normal in *Runx2*^fl/flCre^ mice. However, the phosphorus content in the kidney was increased in *Runx2*^fl/flCre^ mice after a high phosphorus diet, indicating that Runx2 is involved in phosphorus homeostasis at least in high phosphorus diets. 

We previously reported that Runx2 protein and *Runx2* mRNA expressions are downregulated in mature osteoblasts and osteocytes by in situ hybridization and immunohistochemistry [1]. However, real-time RT-PCR analysis showed that *Runx2* mRNA expression in the osteocyte-enriched fraction is higher than that in the osteoblast-enriched fraction. Although we used *Actb* as an internal control in real-time RT-PCR, *Actb* expresses differentially among different cell types [15]. Therefore, the ratio of *Runx2* expression in the osteocyte-enriched fraction against that in osteoblast-enriched fraction does not reflect the ratio of absolute *Runx2* expression. However, we can compare the ratios among *Runx2*, *Galnt3*, and *Fgf23*. The ratios of *Runx2* expression in osteocyte-enriched fraction against that in osteoblast-enriched fraction were higher than those of *Galnt3* expression, indicating that the relative level of *Runx2* expression in osteocytes against that in osteoblasts was higher than that of *Galnt3*. Further, the ratios of *Fgf23* expression were extremely higher than those in *Runx2* and *Galnt3* expressions, indicating that *Fgf23* expression is mostly restricted to osteocytes. As *Fgf23* expression in *Runx2*^fl/flCre^ mice was about half that in *Runx2*^fl/fl^ mice, Runx2 was involved in *Fgf23* expression in osteocytes. Thus, Runx2 regulates *Fgf23* at the transcription level as well as the protein level through the regulation of *Galnt3* expression in osteocytes. 

Increase of the trabecular bone volume in *Galnt3*^−/−^ mice became evident at 30 weeks of age, indicating that the trabecular bone increases during aging. A previous report showed that the femoral trabecular bone volume in *Galnt3*^−/−^ mice was similar to that in the control mice at 8 weeks of age [14]; however, the male *Galnt3*^−/−^ mice, but not female *Galnt3*^−/−^ mice, showed an increase in the femoral trabecular bone volume at 24 weeks of age, although both male and female *Galnt3*^−/−^ mice showed similar serum biochemistries [11]. In our *Galnt3*^−/−^ mice, however, the male and female mice showed similar changes in the trabecular bone at 8 and 30 weeks of age, and the increase of cortical thickness was observed only in female *Galnt3*^−/−^ mice. Although both *Galnt3*^−/−^ mice and *Fgf23*^−/−^ mice showed hyperphosphatemia and hypercalcemia, the bone phenotypes in *Galnt3*^−/−^ mice were quite different from those in *Fgf23*^−/−^ mice. In *Fgf23*^−/−^ mice, osteoid is markedly increased, the mineralized bone volume is severely reduced, BMD is reduced, and the number of osteoblasts and osteoclasts are severely reduced, resulting in low bone turnover [16,17]. In *Galnt3*^−/−^ mice, the osteoid was reduced, mineralized bone volume was increased, BMD was increased or unchanged, and the numbers of osteoblasts and osteoclasts were unaffected, resulting in the normal level of bone formation and bone resorption. The most prominent feature in *Fgf23*^−/−^ mice is the marked increase of osteoid. Fgf23 inhibits phosphate reabsorption by suppressing the expression of type 2a (*Scl34a1*) and 2c (*Scl34a3*) sodium-phosphate cotransporters in renal proximal tubules and reduces 1,25(OH)_2_D_3_ by suppressing the expression of 1α hydroxylase (*Cyp27b1*) [18]. Although both *Scl34a1*^−/−^ mice and *Fgf23*^−/−^*Scl34a1*^−/−^ mice showed hypophosphatemia and hypercalcemia, the marked increase of osteoid was observed in *Fgf23*^−/−^*Scl34a1*^−/−^ mice but not in *Scl34a1*^−/−^ mice [19]. Thus, the serum levels of phosphorus and calcium cannot explain the difference of the osteoid mineralization in *Galnt3*^−/−^ mice and *Fgf23*^−/−^ mice. The serum level of 1,25(OH)_2_D_3_ was markedly increased in *Fgf23*^−/−^ mice and *Fgf23*^−/−^*Scl34a1*^−/−^ mice and mildly increased in *Scl34a1*^−/−^ mice, but not increased in *Galnt3*^−/−^ mice [19]. As an overdose of 1,25(OH)_2_D_3_ inhibits the bone mineralization [20,21], the serum level of 1,25(OH)_2_D_3_, which is regulated by Fgf23, may explain the differential osteoid mineralization in *Fgf23*^−/−^ and *Galnt3*^−/−^ mice. Further, as the serum level of parathyroid hormone (PTH) was undetectable in *Fgf23*^−/−^ mice but one third that of wild-type mice in *Galnt3*^−/−^ mice [19], it may explain the difference of the bone turnover in *Fgf23*^−/−^ mice and *Galnt3*^−/−^ mice. As neither bone formation nor resorption was affected by the absence of *Galnt3*, Galnt3 was unlikely to have a direct effect on osteoblasts or osteoclasts. However, the femoral trabecular bone volume in *Galnt3*^−/−^ mice was increased during aging. Although matrix proteinases (Mmps) including Mmp1, 2, 8, 13, and 14, which are abundantly expressed in bone, cleave type I collagen, the mineralized bone collagen is not attacked by Mmps [22]. Thus, it is suggested that about 60% reduction of the intact Fgf23 in *Galnt3*^−/−^ mice resulted in hyperphosphatemia and hypercalcemia without an increase of serum level of 1,25(OH)_2_D_3_ and without a severe reduction of PTH and led to the accelerated mineralization of osteoid, which inhibits cleavage of type I collagen, and finally to the increase of the trabecular bone volume under the normal bone turnover.

## 4. Materials and Methods

### 4.1. Mice 

*Runx2*^fl/flCre^ mice and *Galnt3*^−/−^ mice were generated as previously described [1,13]. The genetic backgrounds of *Runx2*^fl/fl^ and *Runx2*^fl/flCre^ mice were C57BL/6, and that of *Galnt3*^−/−^ mice was 129Ola/C57BL6. Prior to the investigation, all experimental protocols were reviewed and approved by the Animal Care and Use Committee of Nagasaki University Graduate School of Biomedical Sciences (No. 1403111129-21). Appropriate number of mice were used, and all mice were anesthetized before sacrifice according to the ethical guidelines of animal study. One male mouse was housed per cage and less than three female mice were housed per cage in a pathogen-free environment on a 12 h light cycle at 22 ± 2 °C and 40–70% humidity, with standard chow (CLEA Japan, Tokyo, Japan) and free access to tap water.

### 4.2. Cell Culture

Primary osteoblasts were isolated from the calvariae of wild-type newborn mice by sequential digestion with 0.1% collagenase A and 0.2% dispase. Osteoblastic cells from the third to fifth fraction were pooled and used for cell culture experiments. Cells were plated in 24-well plates at a density of 1.9 × 10^4^ cells/cm^2^ in αMEM supplemented with 10% fetal bovine serum. At confluency, cells were infected with an adenovirus expressing green fluorescent protein (GFP) or *Runx2*-GFP at a multiplicity of infection (MOI) of 10 for 2 h. RNA was extracted after 48 h. A total of 5 × 10^5^ cells were subjected to electroporation with 10 pmol of siRNA for the control or *Runx2* using the Neon Transfection System (Invitrogen, Carlsbad, CA, USA). After 48 h, the medium was changed to an osteogenic medium containing 50 μg/mL ascorbic acid and 10 mM β-glycerophosphate. RNA was extracted after the culture for 7 days in osteogenic medium.

### 4.3. Real-Time RT-PCR Analyses

Muscle, connective tissue, and periosteum were removed from femurs and tibiae of wild-type mice at 9 weeks of age, and the bones were cut at the metaphyses. After hematopoietic cells in the diaphyses of femurs and tibiae were flushed out with PBS, osteoblast-enriched cells were collected using a microintertooth brush (Kobayashi Pharmaceutical Co. Ltd., Osaka, Japan). The remaining bone was used as a source of osteocyte-enriched cells. Total RNA was extracted from the osteoblast-enriched and osteocyte-enriched fractions, whole tibia in *Runx2*^fl/fl^ and *Runx2*^fl/flCre^ mice at 6 weeks of age, and tissues in wild-type mice at 4 weeks of age using ISOGEN (Wako, Osaka, Japan). Real-time RT-PCR was performed using a THUNDERBIRD SYBR qPCR Mix (Toyobo, Osaka, Japan) and Light Cycler 480 real-time PCR system (Roche Diagnostics, Tokyo, Japan). Primer sequences are shown in Supplementary Table S1. We normalized the values obtained to those of *Actb*.

### 4.4. Reporter Assays

A 2.8 kb fragment of the *Galnt3* promoter region was subcloned into the firefly luciferase reporter vector pGL4.10[Luc2] (Promega, Madison, WI, USA) from the BAC clone. All truncated constructs were prepared using the restriction enzyme sites or by PCR amplification. C3H10T1/2 cells were transfected with plasmid DNAs (each luciferase reporter vector 0.1 μg; pRL-Tk Renilla 0.1 μg; pSG5 or pSG5-*Runx2* 0.05 μg) using FuGENE 6 Transfection Reagent (Promega, Tokyo, Japan). Luciferase activities were examined using Dual-Luciferase Reporter Assay System (Promega) and normalized to Renilla luciferase activity.

### 4.5. ChIP Assay

ChIP was performed with a Chromatin Immunoprecipitation Assay Kit (Upstate Biotechnology, Billerica, MA, USA) using the anti-Runx2 monoclonal antibody (Medical & Biological Laboratories, Nagoya, Japan), or mouse IgG (Cell Signaling, Danvers, MA, USA). Immunoprecipitated DNA was amplified using primers in Supplementary Table S1.

### 4.6. Micro-CT Analyses

Micro-CT analysis was performed using a micro-CT system (R_mCT; Rigaku Corporation, Tokyo, Japan). Data from the scanned slices were used for three-dimensional analysis to calculate femoral morphometric parameters. Trabecular bone parameters were measured on a distal femoral metaphysis. Craniocaudal scans of approximately 2 mm (0.2 mm far from the growth plate), for 200 slices in 10 μm increments, were taken. The cortical bone parameters were measured in the mid-diaphysis of the femurs. The threshold of the mineral density was 500 mg/cm^3^.

### 4.7. Serum Test

Blood was collected from the heart and left to stand at room temperature for at least 30 min. Serum was collected after centrifugation at 1500 rpm/min at room temperature for 20 min. The serum levels of calcium, phosphorus, and intact Fgf23 were measured using Calcium E (Dako, Tokyo, Japan), Phosphor C (Dako), and FGF-23 ELISA Kits (Kainos Laboratories, Tokyo, Japan), respectively. The serum level of total osteocalcin was examined using the osteocalcin EIA kit (BTI Biomedical Technologies, Inc., Stoughton, MA, USA) and that of TRAP5b by the TRAcP 5b ELISA kit, Mouse TRAP^TM^ (Immunodiagnostic Systems Ltd., Boldon, UK). 

### 4.8. Measurement of Calcium and Phosphorus in Dry Kidney

*Runx2*^fl/fl^ and *Runx2*^fl/flCre^ mice at 3 weeks of age were fed on high phosphate diet (0.5% calcium and 1.0% phosphorus) for 4 weeks, and they were sacrificed at 7 weeks of age. The kidney samples were dried, ashed, and demineralized with 1 mol/L HCl solution. Ca concentration was analyzed by atomic absorption spectrophotometry (ZA3300, Hitachi High-Tech Corp., Tokyo, Japan) according to the method of Gimblet et al. [23]. P content was measured colorimetrically by the method of Gomori [24].

### 4.9. Statistical Analysis

Values are shown as mean ± SD. Statistical analyses were performed by Student’s *t* test. A *p*-value < 0.05 was considered significant.

## 5. Conclusions

The functions of Runx2 in osteocytes were unclear. *Fgf23* was expressed mostly in osteocytes, and *Fgf23* expression in *Runx2*^fl/flCre^ mice was reduced to half that in *Runx2*^fl/fl^ mice. Thus, Runx2 exerts the functions not only in osteoprogenitors and osteoblasts but also in osteocytes. Runx2 regulated both *Galnt3* and *Fgf23* expressions, but Runx2 was not essential for the phosphorus homeostasis, probably due to a feedback mechanism at the translation level of *Fgf23* mRNA. Although the absence of *Fgf23* markedly increases the serum levels of 1,25(OH)_2_D_3_ and severely reduces PTH, which cause the inhibited mineralization and low bone turnover, respectively, 60% reduction of the intact Fgf23 in *Galnt3*^−/−^ mice did not suppress the osteoid mineralization and bone turnover. Thus, *Galnt3*^−/−^ mice clearly show the effects of hyperphosphatemia and hypercalcemia on bone development and maintenance.

## Data Availability

Data are contained within the article and supplementary materials.

## Supplementary Materials

The following supporting information can be downloaded at: https://www.mdpi.com/article/10.3390/ijms25042275/s1.
