# Supplementary material for: Runx2 Regulates Galnt3 and Fgf23 Expressions and Galnt3 Decelerates Osteoid Mineralization by Stabilizing Fgf23
Source: Int J Mol Sci. 2024 Feb 14;25(4):2275. doi: 10.3390/ijms25042275 (PMC10889289; doi:10.3390/ijms25042275)
Supplement: Supplementary file 1 [file ijms-25-02275-s001.zip › ijms-2826716-supplementary.pdf]

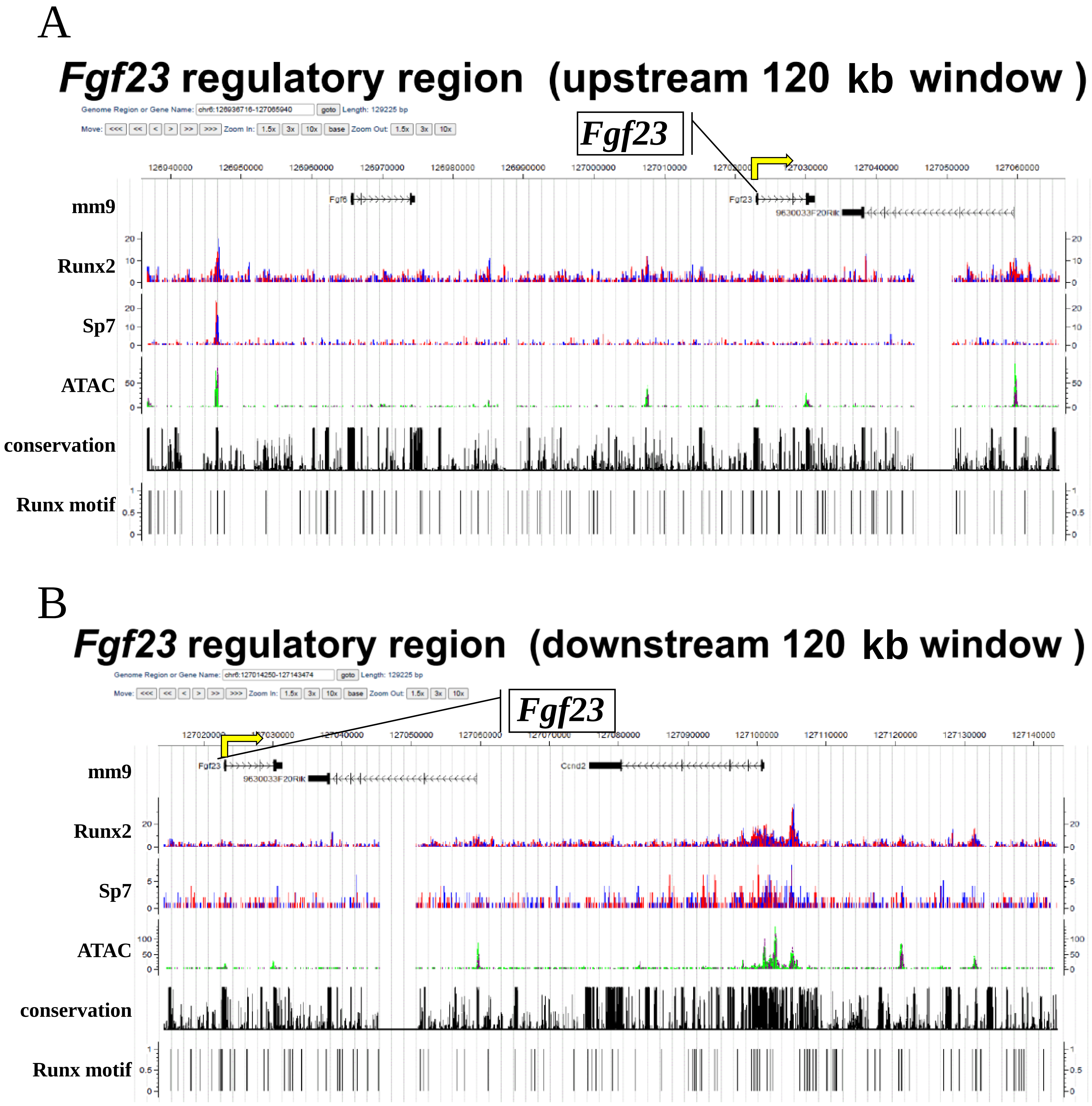

Supplementary Figure S1.

ChIP-seq and ATAC-seq in *Fgf23* locus

CisGenome browser screenshots of the upstream (A) and downstream (B) flanking regions of *Fgf23* showing the association of Runx2 and Sp7 and chromatin accessibility (ATAC) in Sp7-positive primary osteoblasts. The sequence conservation and Runx motif mapping are shown together.

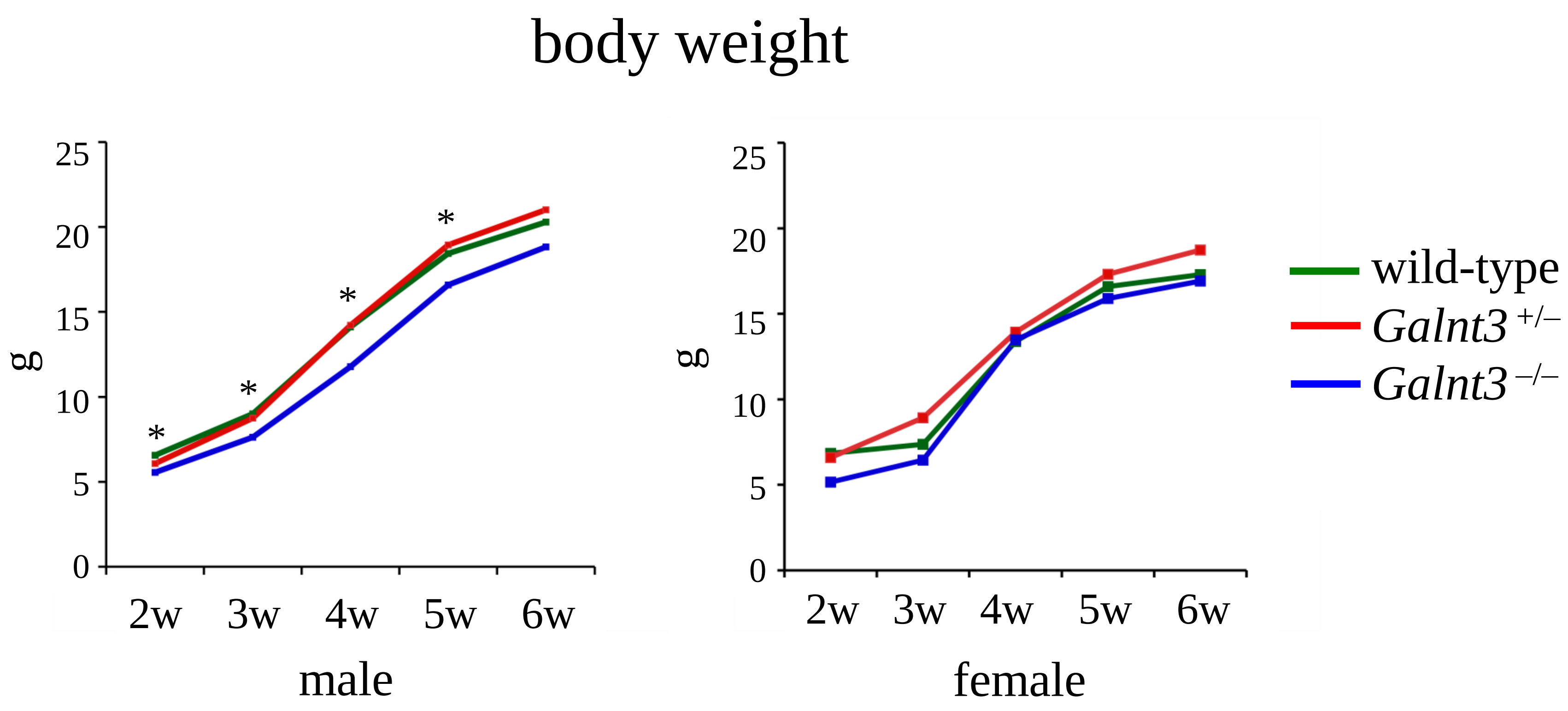

Supplementary Figure S2.

Body weights of male and female wild-type, *Galnt3*$^{+/-}$ and *Galnt3*$^{-/-}$ mice from 2 to 6 weeks of age. n=20-32 per group (male). n=10-21 per group (female). *Versus wild-type mice. *$p < 0.05$.

Supplementary Table S1.

Primer sequences for real-time RT-PCR:

	forward	reverse
	5'-----------------------------------3'	5'-------------------------------------3'
Actb	CCACCCGCGAGCACAGCTTC	TTGTCGACGACCAGCGCAGC
Galnt3	ACACTATTTACCCGGAAGCG	AGCTCCTTCTGGATGTTGTG
Fgf23	ACTTGTCGCAGAAGCATC	GTGGGCGAACAGTGTAGAA
Runx2	AACAAGACCCTGCCCGTG	TGAAACTCTTGCCTCGTCCG
